# Complications Associated With Filler Injection for Breast Augmentation: A Case Report and Literature Review

**DOI:** 10.7759/cureus.95875

**Published:** 2025-11-01

**Authors:** Yingnan Geng, Yiming Gao, Xu Lin, Huaqing Lei, Hengqing Cui, Wenjun Zhang

**Affiliations:** 1 Burns and Plastic Surgery, Second Affiliated Hospital of Naval Medical University, Shanghai, CHN; 2 Plastic and Reconstructive Surgery, Shanghai Ninth People’s Hospital, Shanghai, CHN; 3 Pathology, Second Affiliated Hospital of Naval Medical University, Shanghai, CHN

**Keywords:** breast augmentation, complication, cosmetic, hyaluronic acid, injectable ﬁller

## Abstract

Augmented mammoplasty is a popular cosmetic procedure performed worldwide. Here, we present our treatment experiences with a specific case of complications after a hyaluronic acid injection for breast augmentation to provide constructive advice to clinicians. A 62-year-old female patient was referred to our department with the chief complaint of persistent breast swelling and pain one week following hyaluronic acid injection for augmentation mammoplasty. She underwent two surgical interventions for the removal of necrotic infected tissue and ﬁller, as well as massive irrigation. During the 16-month follow-up period following the second surgery, the patient experienced no complications such as infection, wound dehiscence, or obvious scars. Overall, this case shows that although hyaluronic acid is easy to inject and provides a natural appearance, timely treatment is required once complications occur. Clinicians must focus on the selection of approved fillers with sufficient evidence of safety for use.

## Introduction

Augmented mammoplasty is one of the most popular cosmetic procedures worldwide. The aim of this surgery is to improve women's body image and self-confidence by increasing breast size, perfecting breast shape, and facilitating reconstruction following surgery for breast cancer. With the continuous development of breast filling materials and breast augmentation techniques, an increasing number of surgical methods are available for patients and doctors. While silicone or saline implants are often inserted in both breasts in breast augmentation surgery, selecting the optimal size or shape may be difficult [1]. Moreover, several recent reviews have reported several complications, including asymmetry, implant displacement, and capsular contractures [2].

Fat transplantation is a minimally invasive method of reconstructing the body from autologous tissue, which appears to be highly favored by women seeking cosmetic breast augmentation [3]. However, this procedure is associated with a number of unsightly complications, including the possibility of multiple series of fat injection to achieve the desired breast size, and is not acceptable in cases that request a breast size larger than the original size [4].

Breast augmentation using filler injection has gained popularity in recent years because it causes less trauma and can be performed as a day-care procedures. Initially, a type of injectable substance called polyacrylamide gel was widely used because of its advantages of non-toxicity, stability, and permanency. However, a growing number of complications have been reported [5,6], including mass, infection, pain, migration, and even breast cancer development [7].

In recent years, injectable fillers, such as hyaluronic acid, have been widely used as absorbable fillers to achieve minimally invasive volume enhancement [8]. As a polymer of high molecular weight, hyaluronic acid is a natural component of the body that has a wide range of functions, including dermal fillers, hydrogels, and intradermal injections [9]. Various products comprising hyaluronic acid fillers are available on the market, each with a unique cross-linking degree, concentration, and elastic modulus [10]. Macrolane, a stabilized hyaluronic acid of non-animal origin, was previously a popular product for breast augmentation because of its low risk of allergic reaction or transmission of infectious substances [11,12]. However, it was withdrawn from the market in 2011 due to several reported problems [13].

In this paper, we report the case of a female patient who developed an inﬂammatory reaction at the injection area after undergoing body filler injection for breast augmentation with MAXY FILL, a commercial hyaluronic acid product. The product description for this filler describes it as a Korean filler that can be used for non-surgical body shape correction, and the composition of the preparation was 20 mg/mL. This study aimed to provide constructive advice to clinicians by sharing the treatment experiences.

## Case presentation

**Figure 1:**
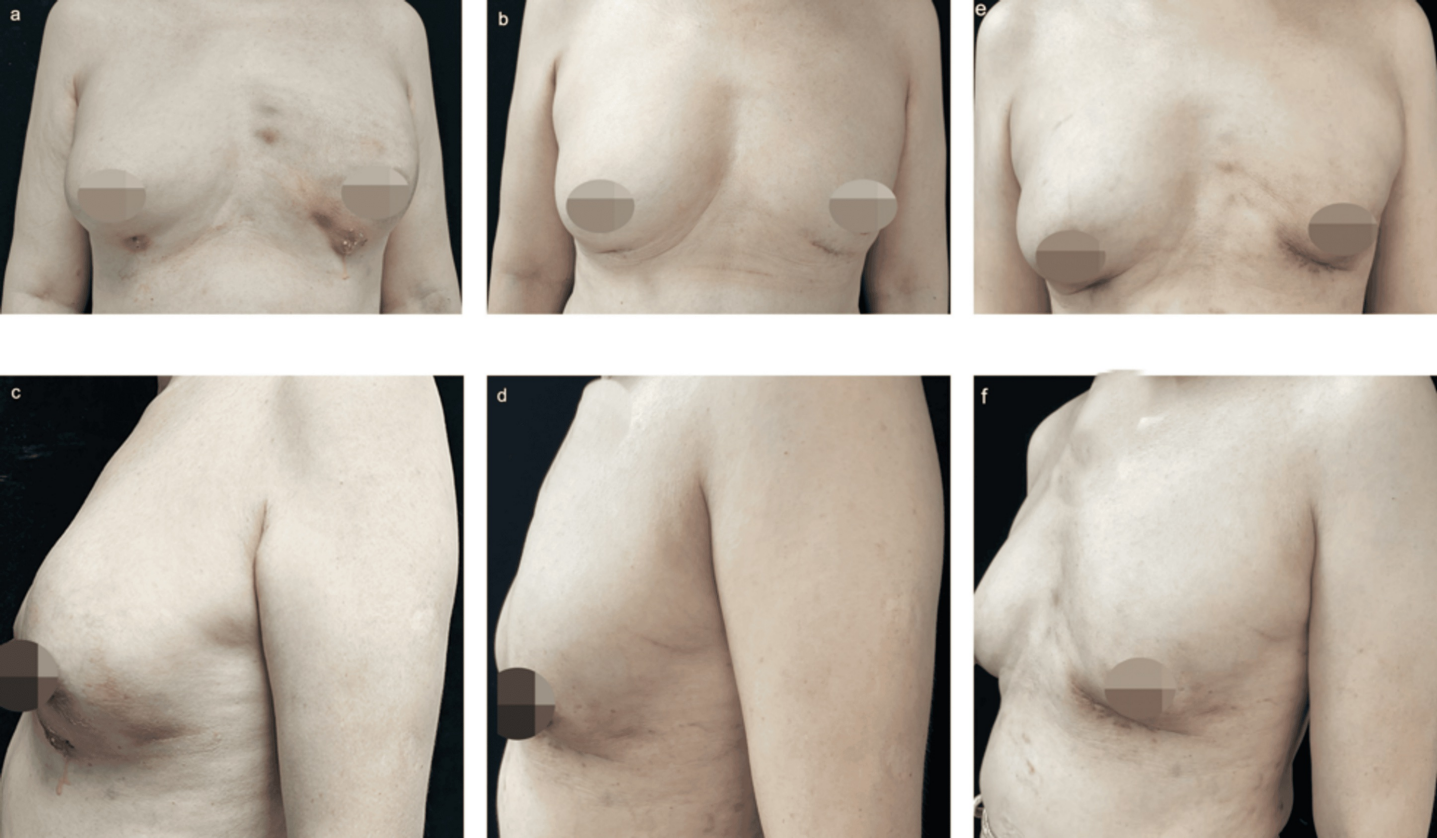
Preoperative and postoperative photographs of both breasts following body filler infection. (a, c) Preoperative photograph of both breasts following body filler infection. A 32-year-old female patient received breast augmentation comprising the injection of the MAXY FILL commercial hyaluronic acid body filler in both breasts. Approximately 60 cc of MAXY FILL body filler and normal saline was injected into each breast. (b, d) Postoperative photograph of both breasts. (e, f) One-month postoperative photograph of the patient who developed a hypertrophic scar. No complications, including infection, wound dehiscence, or obvious scar hyperplasia, occurred during the 16-month follow-up period.

A 62-year-old female patient was referred to our hospital with the chief complaint of persistent breast swelling and pain, followed by liquid outflow from the left breast (Figure 1a). She had undergone breast augmentation comprising injection with a hyaluronic acid product named MAXY FILL in both breasts in a local clinic in Southeast Asia two weeks prior. Approximately 60 cc of MAXY FILL body filler mixed with 60 mL of normal saline was injected into each breast.

**Figure 2:**
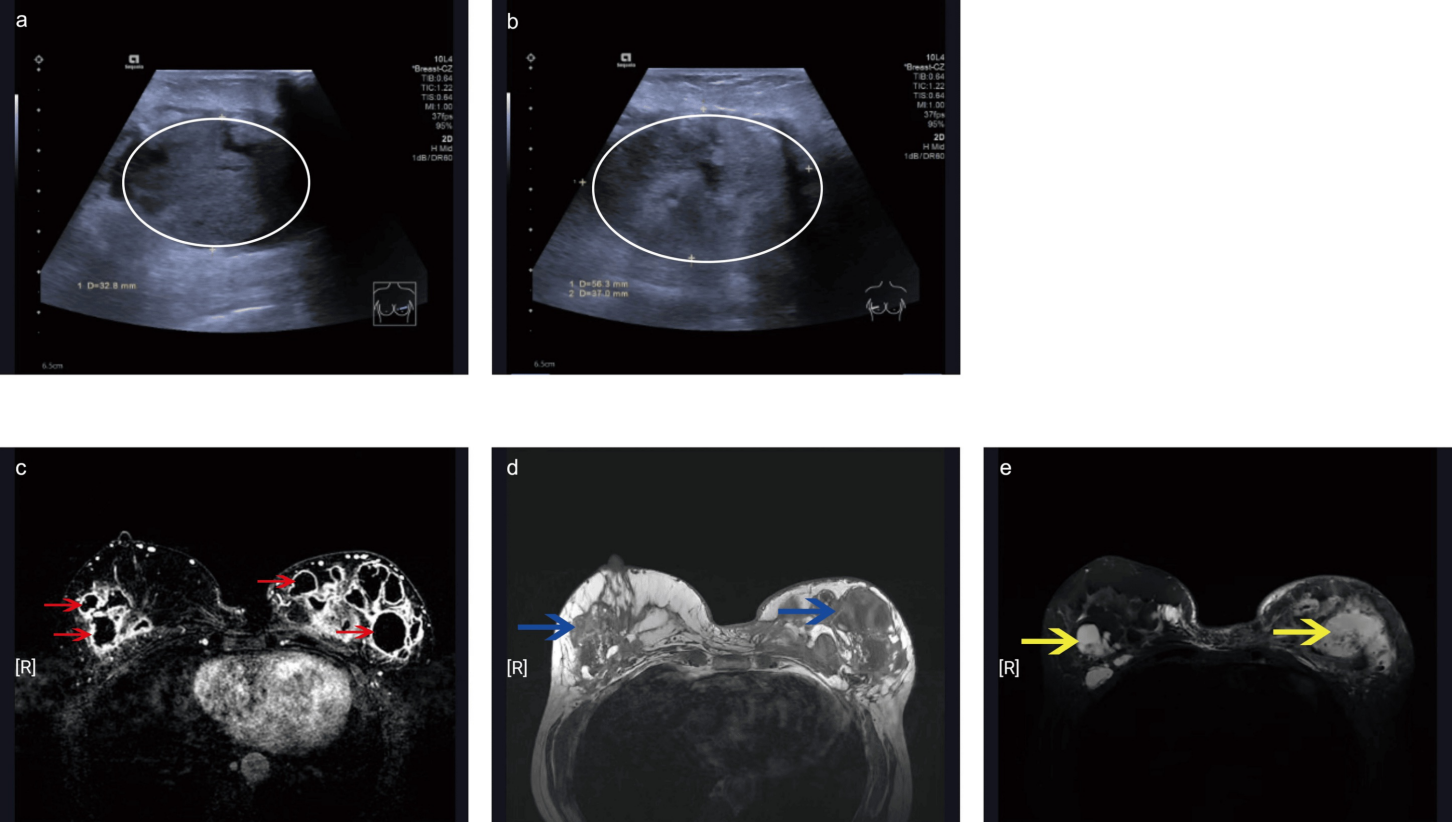
Ultrasonography. Several hypoechoic areas without a clear boundary in the retroglandular space of both breasts (a: left, b: right) suggest abscess formations (ellipse circle). Bilateral breast MRI. (c) Multiple ring-shaped enhancing foci (red arrows) without a clear boundary appear in the retroglandular space, and it was considered as abscess formation. Filler material showing low signal intensity (blue arrows) on fat-saturated T1-weighted (d) and high signal intensity (yellow arrows) on T2-weighted images (e). MRI: magnetic resonance imaging

One week after breast augmentation, the patient developed a high fever and reported redness, swelling, and pain in both breasts. Antibiotic therapy was administered in the outpatient department for seven days, but without significant improvement, and purulent discharge from the left breast was observed. Immediately after admission, culture of secretions from the left breast wound resulted in the growth of methicillin-sensitive *Staphylococcus aureus*. The patient underwent ultrasonography (US) (Figure 2a), which revealed several abscesses without clear boundaries in the retroglandular space of both breasts. Magnetic resonance imaging (MRI) (Figure 2b) was subsequently performed, revealing multiple ring-shaped enhancing foci without clear boundaries in the retroglandular space, which was consistent with an abscess formation. Filler material was observed as low signal intensity on fat-saturated T1- and high signal intensity on short-tau inversion recovery (STIR) T2-weighted images.

**Figure 3:**
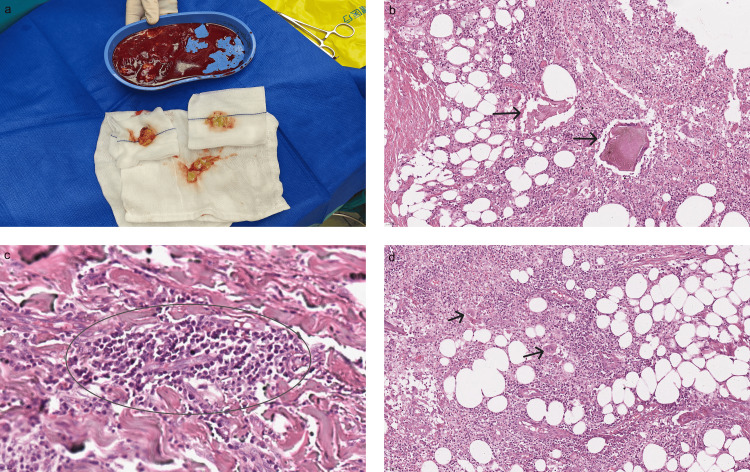
Pathology results of the filler and necrotic tissue (hematoxylin and eosin (HE) stain, ×100). (a) Intraoperatively, partial body filler and necrotic tissue were removed from the incisions of both breasts. (b) Diffuse distribution of the body ﬁller (arrow). (c) Subcutaneous neutrophil microabscess formation represents acute inflammation (circle) and (d) foreign body giant cell (arrow).

The final diagnosis was bronchoglandular tissue infection resulting from the filler injection. The patient underwent an initial operation to remove the filler and necrotic tissue through the skin ulceration site on the inframammary fold. The filler and necrotic tissues (Figure 3a) were removed by scraping and flushing the purulent cavity with large amounts of hydrogen peroxide, iodophor, and chlorhexidine solution. A negative-pressure drainage tube was inserted into each breast vomica. The incision was not sutured, and the wound was filled with gauze and pressure bandaged with a sterile dressing. Dressing treatment was administered daily. Intravenous antibiotics sensitive to methicillin-sensitive *S. aureus* were administered for seven consecutive days postoperatively. Pathology results suggest the diffuse distribution of the body ﬁller (Figure 3b) and subcutaneous neutrophil microabscess formation representing acute inflammation (Figure 3c) and foreign body giant cell (Figure 3d). To drain the possible residual necrotic tissue and purulent secretions, we kept the incision open without suturing or closure. As in the prior surgery, the cavities in both breasts were scraped and irrigated with hydrogen peroxide, iodophor, or normal saline during the second procedure. Two indwelling drains were maintained for 5-7 days, and the wound was closed using sutures after achieving exact hemostasis. The patient developed a hypertrophic scar at the incision site in the early stage of healing. After receiving intense pulsed light treatment and a subsequent carbon dioxide laser treatment within half a year, there was a significant improvement. No complications, including infection, wound dehiscence, or obvious scar hyperplasia, occurred during the 16-month follow-up period (Figure 1b). All procedures performed in studies involving human participants were in accordance with the ethical standards of the institution and with the 1964 Helsinki declaration and its later amendments or comparable ethical standards.

## Discussion

Breast augmentation with hyaluronic acid injection is a safe, non-surgical, and effective method to enhance the size and shape of the breast. As with any other filler, hyaluronic acid must be injected under sterile conditions to ensure sterility. Once an infection occurs, management is complicated, and relapses readily occur [14,15]. Common complications of hyaluronic acid breast augmentation include infection, filler displacement/nodules, early degradation, and breast hardening, which can be managed with antibiotics, hyaluronidase dissolution, or surgical drainage [12,13]. Ultrasound-guided injection and strict aseptic operation can reduce the risk, but regular maintenance and vigilance against imaging interference are necessary [16]. Severe complications (such as lymph node metastasis) require surgical intervention [10]. The infection in our case was probably due to failure to use an aseptic technique during injection, resulting in infection and abscess formation. Injection of a mixture of hyaluronic acid filler and saline may also have increased the risk of contamination. Fortunately, bacterial cultures of secretions were performed immediately after the patient’s admission, and sensitive antibiotics were selected to resist infection according to the results of the drug sensitivity test.

Preoperatively, both US and MRI revealed multiple abscesses in the breast. MRI is the preferred method for assessing the integrity of breast implants and complications [17]. To drain the possible residual necrotic tissue and purulent secretions, we kept the incision open without suturing or closure. Once obvious wound exudation had been halted, a second surgery was performed to close the wound.

Generally, there are two choices of incisions: along the rim of the areolar and inframammary folds. We chose the rupture site of the breast as an incision, which was close to the inframammary fold, to facilitate the opening of the purulent cavity directly and postoperative drainage and to reduce the formation of another scar. Overall, we present this case as an example to provide constructive advice to clinicians by sharing our treatment experience.

## Conclusions

This case report describes a patient who developed complications after hyaluronic acid injection for augmentation mammoplasty. Although hyaluronic acid is easy to inject and provides a natural appearance, timely treatment is required once complications occur. Currently, various injection fillers of varying quality are available on the market, and a growing number of complications occur after hyaluronic acid filling to achieve the desired effect, especially infection. To prevent local adverse events, sterile treatment is required. Early treatment of local adverse reactions should also be implemented. Therefore, clinicians must focus on selecting approved fillers with sufficient safety evidence for use and strictly follow the aseptic technique and the guidelines of infection control during the injection procedure.
